# An Evaluation of Patient and Student Experience at a Longstanding Student-run Free Clinic in Cape Town, South Africa

**DOI:** 10.7759/cureus.6320

**Published:** 2019-12-07

**Authors:** Madeleine Heller, Alexandria M Thomas, Shrikant M Peters, Kira M Düsterwald, Jeffrey D Klausner

**Affiliations:** 1 Medicine, David Geffen School of Medicine at University of California - Los Angeles, Los Angeles, USA; 2 Medicine, Charles R. Drew University of Medicine and Science, Los Angeles, USA; 3 Public Health Medicine, University of Cape Town and Groote Schuur Hospital, Cape Town, ZAF; 4 Human Biology, Faculty of Health Sciences, University of Cape Town and Groote Schuur Hospital, Cape Town, ZAF

**Keywords:** student-run free clinics, medical education, patient satisfaction

## Abstract

Background

Student-run free clinics (SRFCs) combine medical student learning with the provision of free health care. A comprehensive evaluation of patient experience at SRFCs is needed to ensure a balance between valuable clinical experience for students and enhancement of patient care. The aim of this study was to describe patient and medical student perception of care at a longstanding SRFC at the University of Cape Town (UCT).

Methods

We conducted an observational study at the Students' Health and Welfare Centres Organisation (SHAWCO), a student-run free clinic at UCT. Trained study staff observed clinical encounters between consenting medical students and patients. We surveyed patients on their demographic characteristics, overall satisfaction, and impressions of medical students and physicians at SHAWCO. We surveyed medical students on their level of training, motivation for volunteering, and future career plans. We linked all data from each clinical encounter by a study-generated identification number.

Results

We surveyed a total of 34 patients and 52 medical students on their experience at SHAWCO. All patients either strongly agreed (88%) or agreed (12%) that they were satisfied with care. Patient satisfaction did not vary with the parameters of care included in multivariable analysis. Patients rated medical students higher than physicians on listening skills, and equally to physicians on all other clinical skills rated. Medical students reported a strong desire to go into primary care and work in underserved settings both before and after volunteering at SHAWCO.

Discussion

We found a high level of patient satisfaction at SHAWCO, consistent with other studies. Our findings indicate that medical student involvement in care at SRFCs is not a detriment to patient satisfaction.

## Introduction

Student-run free clinics combine student learning with the provision of free health care. Over 100 student-run free clinics (SRFCs) are currently in operation at US medical schools and greater than 36,000 annual patient-physician visits at SRFCs were reported in 2007 [1,2]. At SRFCs, a student often performs the initial patient evaluation before presenting the case to a qualified clinician. Past studies have shown SRFCs to be a feasible and effective means of providing care to uninsured, poverty-stricken, or otherwise vulnerable populations [3-6].

In a survey of 86 student-run clinics around the US, students identified student education and providing care in underserved communities as their clinics’ greatest strengths [1]. SRFCs encourage students to learn what resources are available to inner city populations and where there are gaps in services [7]. While students report personal and professional benefit from volunteering at SRFCs, further investigation is needed to understand whether SRFC involvement increases students’ likelihood of practicing in underserved areas after medical school. Studies have shown that medical students volunteering at SRFCs have a high level of intrinsic motivation, but have yet to define these intrinsic motivators [8].

Most studies evaluating patient satisfaction at SRFCs have shown high satisfaction with care provided [6,9,10]. However, the patient perspective of care received at student-run free clinics has only recently been explored in a small number of studies, most taking place in affluent nations [10-13]. A recent South African study from Du Toit et al. found high patient satisfaction with a mobile clinic for community-based service learning at Nelson Mandela University in the Eastern Cape province [9]. Overall, little is known about how organizational and service differences improve or hinder patient satisfaction at SRFCs versus other care models. More comprehensive evaluation of patient experience at SRFCs is needed to ensure a balance between valuable clinical experience for students and enhancement of patient care.

The aim of this study was to better understand what makes a student-run free clinic worthwhile for patients, understand how the clinical encounter in an SRFC is perceived by both patients and medical students, and identify interventions that may be implemented to improve clinic efficacy. Students' Health and Welfare Centres Organisation (SHAWCO) health clinics were chosen as the model SRFC. A secondary aim was to understand whether the SHAWCO health, a student-run free clinic at the University of Cape Town, has an impact on the career goals and/or specialty choice of participating medical students.

## Materials and methods

We used a mixed methods approach to evaluate patient satisfaction with care received at the Student Health and Welfare Centres Organisation (SHAWCO) Health clinics at the University of Cape Town School of Medicine [14]. SHAWCO is the oldest student-run free clinic in South Africa and serves over 4,000 patients a year. We surveyed SHAWCO patients on their perception of care providers at SHAWCO and overall satisfaction with the visit. Secondly, we surveyed medical students volunteering at SHAWCO on their prior clinical experience, confidence, and motivations for volunteering. We linked data from patient-medical student pairs (from a single clinical encounter) by study-generated identification numbers. In cases where both the medical student and patient from a single encounter consented to participate, we observed the clinical encounter and recorded objective measures of care such as language concordance, medical student interpersonal/clinical practices, and diagnostic studies performed. If multiple students from a single case were surveyed, we used data from the senior-most student in any combined analyses of patient and student responses.

Patient data collection

We recruited SHAWCO patients over the age of 18 years and not in urgent need of medical care at SHAWCO sites in Simthandile, Du Noon, Hout Bay, and Masiphumelele between May 25 and July 15, 2018. We provided consenting patients with an information sheet discussing the aims, risks, and benefits of the study. Study co-investigators or trained staff then consented and enrolled those wanting to participate. Patients completed a survey after the consultation.

We based our Patient Satisfaction Survey on the South African Human Sciences Research Council (2008) version of the Health Systems Trust Client Satisfaction Survey Questionnaire and other patient satisfaction surveys administered in the US. We reviewed these survey tools with local providers and updated them to fit our study aims. Surveys assessed demographic characteristics, accessibility, patient motivations for attending SHAWCO, patient-provider interactions, and patient overall satisfaction.

Medical student data collection

Co-investigators and study staff recruited medical students over 17 years old and actively involved in care provision at SHAWCO. We provided consenting students with an information sheet discussing the aims, risks, and benefits of the study. Study co-investigators or trained staff then consented and enrolled those wanting to participate. The medical student questionnaire assessed the student’s experience and perception of patient satisfaction. The survey was based on the Confidence survey previously administered on medical students to assess future career choice [15]. Questions were modified to fit our study objectives, within the context of a South African student-run free clinic. We assessed demographic characteristics, student satisfaction with the care provided, opinion of the patient’s overall satisfaction, the impact of volunteering on students’ specialty choice/career plans, and motivation for volunteering.

Clinical encounter observation

During the clinical encounter, trained study staff recorded care team clinical practices/competencies and visit outcomes on a pre-formed checklist.

Statistical concerns

Trained study staff recorded survey response options for patients and medical students on Likert Scale, as binary responses, or as short answer responses. Our target sample size was 100 SHAWCO patients and 100 student participants for a desired margin of error of 10%.

We determined frequencies, means, and standard deviation of patient data with Excel Pivot Table. We performed paired sample t-tests in Stata IC (College Station, TX: StataCorp LP) to identify any difference in patient satisfaction with medical students and physicians. We ran a linear regression in Stata to identify any factors adjusted associations with patient satisfaction. We deemed p values of less than 0.05 significant.

We included any unpaired patient data in analyses of patient satisfaction unrelated to medical student characteristics. We used any unpaired data from student surveys in analyses of medical student experience unrelated to patient satisfaction.

Funding source

The David Geffen School of Medicine Short Term Training Program, UCLA Center for World Health, Team Klausner Saving Lives, and UCLA Center for AIDS Research (CFAR) NIH/NIAID AI028697 provided financial support for this study.

Human subjects

The Institutional Review Board at the University of California Los Angeles and the University of Cape Town Departmental Research Committee at the School of Public Health approved this study.

## Results

We surveyed 34 patients and 52 medical students, and observed a total of 35 clinical encounters at SHAWCO Health sites. We collected a complete set of patient and medical student survey data from 30 clinical encounters.

Patient and medical student participant demographic characteristics

Patient demographic characteristics are represented in Table 1. Medical student demographic characteristics are presented in Table 2. Of 34 patients completing surveys, 33 (97%) traveled by foot to reach clinic. Twenty-seven (93%) patients reported traveling under 30 minutes and two (7%) greater than 30 minutes to arrive at the SHAWCO site.

**Table 1:** Demographic characteristics of patients at Student Health and Welfare Centres Organisation (SHAWCO) health clinics in Cape Town, South Africa (May 25–July 15, 2018). A: Participants were able to select “Other”. B: Not all n values are equal to 34 as patients were allowed to skip questions freely.

Patients	
Sex, n	34^A^
Male	6 (17.6%)
Female	28 (82.4%)
Age, n	33^B^
20-39	4 (12.1%)
30-39	13 (39.4%)
40-49	10 (30.3%)
50+	6 (18.2%)
Primary Language, n	32^B^
isiXhosa	26 (81.3%)
Afrikaans	4 (12.5%)
Other^B^	2 (6.3%)
Employment Status, n	34
Full time	19 (55.9%)
Part time	7 (20.6%)
Looking for work	5 (14.7%)
Self employed	0 (0%)
Other	3 (8.8%)
Education Level Completed, n	28^B^
Greater than grade 7	24 (85.7%)
Grades 1-7	4 (14.3%)

**Table 2:** Demographic characteristics of medical students surveyed at Student Health and Welfare Centres Organisation (SHAWCO) health clinics in Cape Town, South Africa (May 25–July 15, 2018). A: Participants were able to select “Other”.

Medical students	
Sex, n	52^A^
Male	25 (48.1%)
Female	27 (51.9%)
Year in school, n	52
1^st^ year	1 (1.9%)
2^nd^ year	0 (0%)
3^rd^ year	19 (36.6%)
4^th^ year	14 (26.9%)
5^th^ year	5 (9.6%)
6^th^ year	13 (25%)
Primary language, n	52
English	33 (63.5%)
isiXhosa	5 (9.6%)
Afrikaans	2 (3.8%)
Other	12 (23.1%)
Frequency Volunteered, n	52
Once	10 (19.2%)
2-5 times	12 (23.1%)
6-10 times	2 (3.8%)
11-20 times	6 (11.5%)
Over 20 times	22 (42.3%)

Patient satisfaction

All patients either strongly agreed (88%) or agreed (12%) that they were satisfied with the care they received at SHAWCO. Patient-reported ratings of medical students and physician clinical skills are presented in Table 3.

**Table 3:** Comparison of mean patient-reported score of medical student and physician competencies at Student Health and Welfare Centres Organisation (SHAWCO) health clinics in Cape Town, South Africa (May 25-July 15, 2018). A: Patients were asked to rank medical students and physicians on the same parameters after the encounter. A paired sample t test was performed to assess differences in patient perception of medical student and physician skill.

	Mean (SD) of medical student score	Mean (SD) of physician score	P value^A^
Listened to my concerns	4.70 (0.618)	4.48 (0.906)	0.018
Respected my privacy	3.50 (1.796)	3.44 (1.812)	0.871
Comfort in asking questions about health	4.00 (1.436)	3.94 (1.516)	0.692
Knowledgeability	4.41 (0.988)	4.60 (0.783)	0.245
Display of dignity and respect	4.85 (0.359)	4.79 (0.410)	0.325

Medical student demographic characteristics and experience

A total of 52 medical students were surveyed, including 33 senior medical students and 19 junior medical students. The demographic characteristics of medical students surveyed are shown in Table 2.

**Figure 1:**
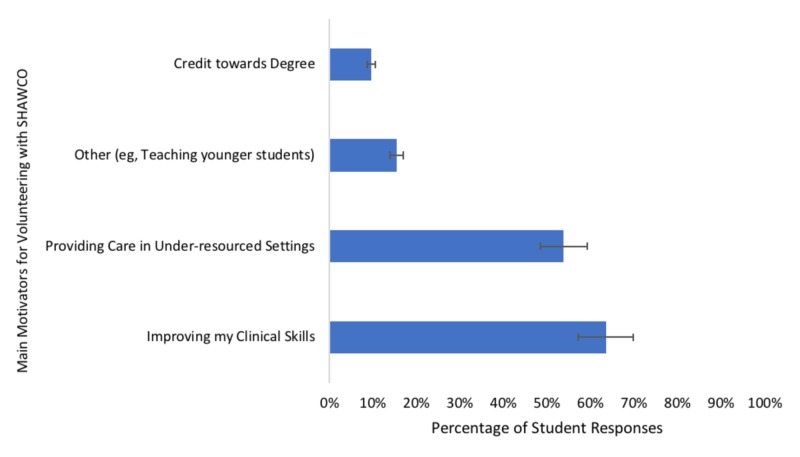
Medical student self-reported motivations for volunteering with Student Health and Welfare Centres Organisation (SHAWCO) health clinics in Cape Town, South Africa (May 25-July 15, 2018).

Student-reported motivations for volunteering at SHAWCO Health clinics are shown in Figure 1.

**Figure 2:**
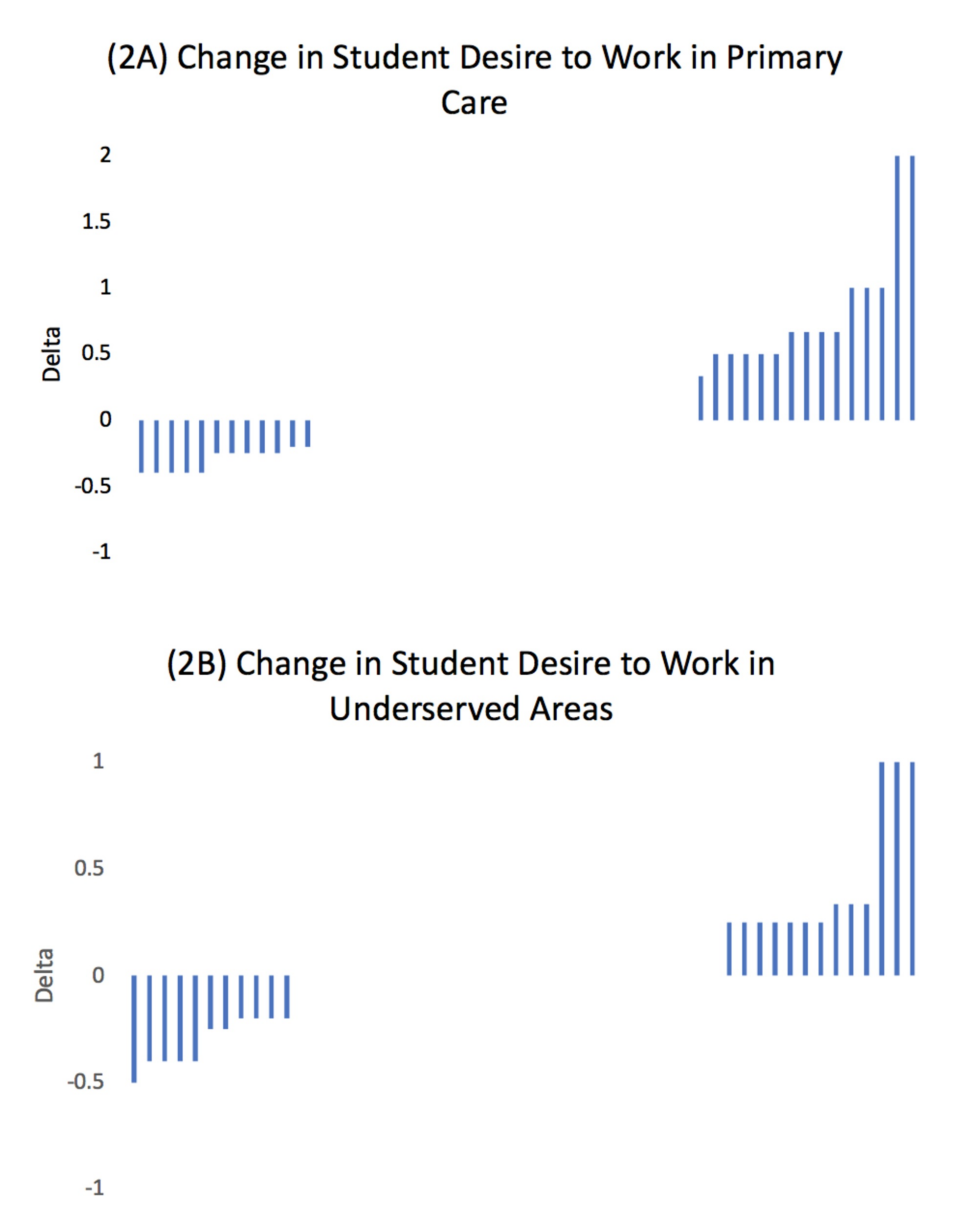
Change in medical student desire to work in primary care (A) and underserved areas (B) before and after volunteering with Student Health and Welfare Centres Organisation (SHAWCO) health clinics (May 25-July 15, 2018). Each column represents one of the 52 students surveyed. Students were asked to rate their desire to work in primary care or underserved settings before and after volunteering at SHAWCO on a Likert Scale of 1-5 (1 = lowest desire, 5 = highest desire). Of surveyed students, 25/52 reported no change in desire to work in primary care and 28/52 students reported no change in desire to work in underserved settings. Positive delta values indicate increased desire among students after volunteering at SHAWCO.

Change in individual students’ desire to work in primary care and under-resourced settings is shown in Figure 2A-2B. The mean impact of volunteering at SHAWCO on student-reported desire to work in primary care before volunteering was 3.3 and after volunteering was 3.5 on a Likert scale (0 = lowest rating, 5 = highest rating). The mean impact of desire to work in underserved areas before and after volunteering was 3.9 on a Likert scale. Mean medical student desire to work in primary care or under-resourced settings by year of training did not change before and after volunteering at SHAWCO (p = 0.5004 and 0.8647, respectively).

Clinical encounter findings

Medical student competencies/clinical practices and clinical outcomes of the encounter (testing, treatment) are represented in Table 4.

**Table 4:** Medical student skills and clinical encounter outcomes at Student Health and Welfare Centres Organisation (SHAWCO) health student-run free clinic at the University of Cape Town, South Africa (May 25-July 15, 2018). A: Frequencies do not add up to 100 as study staff recorded all options that applied to the case.

	Number (percent) of student-patient encounters
Medical student speaks patient's primary language fluently (n = 36)	27 (75%)
Medical students introduced themselves to patient (n = 36)	34 (94%)
Medical student asked patient if they had any questions	21 (63%)
Medical student method of addressing patient (n = 20)	
Asked patient what they like to be called	6 (30%)
Confirmed that name on SHAWCO form was correct	6 (30%)
Assumed what patient would like to be called (i.e., “sisi”)	4 (20%)
Did not address patient by name during encounter	4 (20%)
Patient received point-of-care testing: (n = 39)	30 (88%)^A^
Blood pressure	27 (69%)
Urinalysis	12 (31%)
Blood glucose	10 (26%)
HIV	4 (10%)
Pregnancy	4 (10%)
Patient received treatment: (n = 34)	26 (76.4%)^A^
Body/limb aches	8 (21%)
Coughing	7 (18%)
Headache	7 (18%)
Fever	4 (10%)
Rash	4 (10%)
Diarrhea	1 (3%)
Patient received referral form to other health care site (n = 34)	18 (52.9%)

Impact of various aspects of care at SHAWCO on patient satisfaction

On multivariate analysis, patient satisfaction was not associated with language concordance (Coef. = 0.133, 95% CI [-0.168, 0.435], p = 0.368) whether or not the medical student introduced themselves to the patient (Coef. = 0.016, 95% CI [-0.410, 0.443], p = 0.936), medical student year of training (Coef. = 0.012, 95% CI [-0.092, 0.116], p = 0.814), medical student self-reported confidence in taking a history (Coef. = -0.088, CI [-0.221, 0.045], p = 0.185) or performing a physical exam (Coef. = 0.087, 95% CI [-0.099, 0.273], p = 0.343), sex of patient (Coef. = -0.180, CI [-0.135, 0.494], p = 0.248) or patient employment status (Coef. = -0.021, CI [-0.110, 0.069], p = 0.633).

Insufficient data were collected to evaluate the impact of the following factors on patient satisfaction: medical student motivation for volunteering at SHAWCO, clinical experience of medical student, whether the medical student asked permission to examine the patient/asked if the patient had questions, how the medical student addressed the patient, patient age, and patient primary language.

## Discussion

We completed a survey of patients and medical students at SHAWCO Health, a student-run free clinic at the University of Cape Town School of Medicine. Patients at SHAWCO reported a high level of satisfaction with the care provided. We found that the most common motivator for medical student involvement in SHAWCO was improving clinical skills, followed by providing care in an under-resourced setting. Furthermore, students did not report a change in desire to work in primary care or under-resourced settings before and after volunteering at SHAWCO.

Consistent with our findings at SHAWCO, several studies have found high patient satisfaction ratings of student-run free clinics [6,9-11]. Interestingly, SHAWCO patients rated medical students higher than physicians on listening to their concerns. This highlights a niche role for students on a patient care team. In a study conducted at the University of Central Florida KNIGHTS clinic, Lu et al. found that patients identified free/affordable medical care, primary/specialty care, and the team approach as the greatest strengths of SRFCs [10]. Based on our results, the presence of students at SRFCs may enhance patient care by adding a layer to the “team approach.” Patients surveyed in our study perceived medical students as superior listeners, fulfilling a role known to impact patient satisfaction. Forbes and Nolan found that student communication was associated with patient satisfaction at a student-led physiotherapy clinic [12]. While the physician must see many patients in a short amount of time, the student can listen to the patient's concerns more extensively and summarize them to the rest of the team. In short, the presence of a medical student may improve patients’ sense of being heard at clinic.

In our study, patients rated medical students and physicians equally on respecting privacy, creating a comfortable environment for the patient to ask questions, being knowledgeable, and displaying dignity and respect. Overall, medical student involvement at SRFCs did not have a negative impact on patient trust or confidence in the care received. Patient confidence in the care team is an important aspect of adherence, follow-up, and overall treatment [15].

Patients rated respect for privacy the lowest of all the measures for both medical students and physicians. Unfortunately, student-run free clinic encounters are often conducted in open spaces with little privacy for patients during the history and exam. At SHAWCO, most evaluations are conducted in a sub-divided trailer with sliding doors between consultation rooms. High overall satisfaction ratings in our study imply that lack of privacy did not greatly impede patient satisfaction. This may be indicative of patients having few other treatment options or having accepted lack of privacy in medical care as a norm. Increasing privacy for patients at SRFCs may improve patient experience.

Understanding medical student motivation for volunteering at SRFCs is valuable for optimizing recruitment and retention of students into their clinical years. Further, understanding medical student experience at SRFCs is important in encouraging students to go on to work with vulnerable populations as physicians. Similar to other studies evaluating student experience, our findings demonstrate that volunteering at SRFCs does not impact student desire to work in primary care or underserved settings in the future [16,17]. Medical students choose to volunteer at SHAWCO in addition to their core curricular activities and are not required to participate. As a result, students choosing to volunteer at SHAWCO during their time at University of Cape Town (UCT) are already highly motivated to work in underserved areas and/or as primary care physicians, making it difficult to observe changes in career motivation. We described intrinsic motivators for volunteering amongst medical students at SHAWCO Health clinics, including improving clinical skills and providing care in under resourced areas. Interestingly, several students reported in the “other” category that the opportunity to teach their peers in the years below them was a motivator for attending clinic. SRFCs that struggle to recruit more advanced students to clinics may benefit from promoting the opportunity to teach at clinic. It is important to note that, in our study, student’s desire to work in underserved areas was high, at neutral or greater, both before and after volunteering and students invested in SRFCs should be sought after when looking for future primary care clinicians or clinicians who want to work in underserved settings.

A thorough understanding of the medical student’s role on the care team is critical for training students, planning clinic operations, and ultimately maximizing patient experience at student-run free clinics. Ideally, we would conduct a longitudinal study to see if these experiences increase the number of students entering primary care, preventive community health or family medicine residencies or working in underserved areas. Future studies are also needed to evaluate patient experience at SRFCs in comparison with a control group of patients and care providers at a non-student-run clinic in a similar setting. Our findings are limited in that all data was collected at a single time point, with students asked to report past sentiments that may be misremembered. Other limitations of this study include a small sample size due to a short time course of data collection. Additionally, this study addresses only the patient viewpoint of the clinical encounter at SHAWCO. Future evaluation of SRFCs should also include assessment of health outcomes, loss to follow up, and patient perception of care at SRFCs versus other health care options.

## Conclusions

Patients at SHAWCO Health have a high level of satisfaction with the care provided at SHAWCO. Medical students are perceived as superior listeners and therefore serve an important role on the care team. At baseline, medical students involved in SHAWCO have a high level of motivation to pursue primary care and work in underserved areas. Direct comparison of student-run free clinics with non-student-run clinics is needed to fully understand the impact of student involvement and leadership on patient experience.
